# Anisotropic Electrostatic Interactions in Coarse-Grained Water Models to Enhance the Accuracy and Speed-Up Factor of Mesoscopic Simulations

**DOI:** 10.1021/acs.jpcb.1c07642

**Published:** 2021-10-27

**Authors:** Francesco Maria Bellussi, Otello Maria Roscioni, Matteo Ricci, Matteo Fasano

**Affiliations:** † Department of Energy, Politecnico di Torino, Torino 10129, Italy; ‡ MaterialX LTD, Bristol BS2 0XJ, U.K.

## Abstract

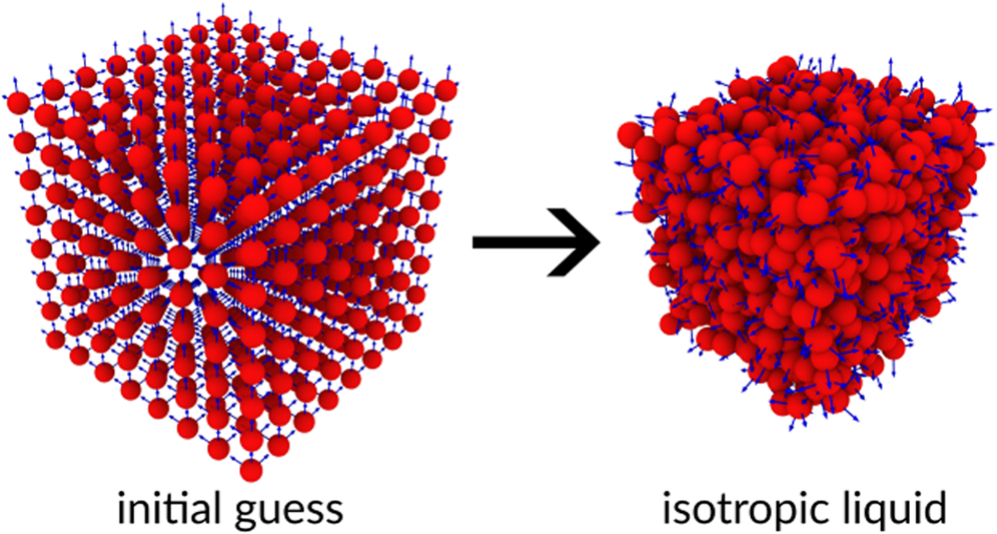

Water models with realistic physical–chemical properties are essential to study a variety of biomedical processes or engineering technologies involving molecules or nanomaterials. Atomistic models of water are constrained by the feasible computational capacity, but calibrated coarse-grained (CG) ones can go beyond these limits. Here, we compare three popular atomistic water models with their corresponding CG model built using finite-size particles such as ellipsoids. Differently from previous approaches, short-range interactions are accounted for with the generalized Gay–Berne potential, while electrostatic and long-range interactions are computed from virtual charges inside the ellipsoids. Such an approach leads to a quantitative agreement between the original atomistic models and their CG counterparts. Results show that a timestep of up to 10 fs can be achieved to integrate the equations of motion without significant degradation of the physical observables extracted from the computed trajectories, thus unlocking a significant acceleration of water-based mesoscopic simulations at a given accuracy.

## Introduction

Coarse-grained (CG) molecular dynamics (MD) simulations offer an efficient way to study the most diverse systems at a mesoscopic scale, with applications ranging from biochemistry^1,2^ to material science.^3−5^ The basic idea behind CG is to decrease the number of interacting sites describing individual molecules. By reducing the model resolution, the computational cost and the configuration space of the system decrease, thus enabling the modeling of larger and more complex systems compared to atomistic simulations. For some phenomena, such as the conformation change of enzymes and functional proteins,^6,7^ the limiting factor of all-atom (AA) simulations is the timescale needed to witness a specific process. In this regard, CG models enable longer timesteps and thus accessible simulation times by suppressing the high-frequency motion characteristics of light atoms and/or averaging out some intramolecular degrees of freedom. Several CG models of water have been proposed during the last two decades, following different approaches for the mapping process.^8−12^ However, current CG models often do not provide an adequate description of intermolecular interactions due to the lack of many-body contributions, which are particularly important for the accurate description of, for example, water properties.^13^ Instead, the MOLC model uses finite-size aspherical particles^14^ connected with directional bonds. The particles are decorated with virtual sites representing point charges. Differently from previous approaches, short-range interactions are accounted for with the generalized Gay–Berne potential,^15^ while electrostatic and long-range interactions with a modified version of the usual Coulomb pairwise summation and the reciprocal-space Ewald solver, respectively.^16^

This work explores the tantalizing possibility of carrying out CG-MD simulations of liquid water with near-atomistic quality using the MOLC model,^16^ which is available as a user-defined package for the popular materials modeling code LAMMPS.^17,18^ The CG models of water presented in this work use one site to represent the whole molecule but with the possibility to host an arbitrary number of virtual charges to account for realistic electrostatic interactions. Other one-site water models were described in the literature, e.g., using a Stillinger–Weber potential to mimic the effect of anisotropic hydrogen-bonding interactions,^19^ projecting the many-body interactions into pairwise basis sets,^20^ or accounting for electrostatic interactions with a point dipole,^10^ even if significant discrepancies with AA models have been shown. However, many biological processes such as electropore formation in membranes are governed by electrostatic interactions, and thus more detailed modeling of electrostatics is required to capture the dynamic behavior of water.^21^ Our solution is to explicitly include three virtual charges per water molecule, having a 1-to-1 correspondence between the CG model and the source AA model. Hence, the aim of this work is to test that atomistic models of water can be rewritten in terms of finite-size spheres (thus taking advantage of the longer timesteps allowed by the Richardson iteration method^22^), without significantly losing the accuracy of all-atom description for structural and dynamical properties of liquid water.

## Computational Methods

**Figure 1:**
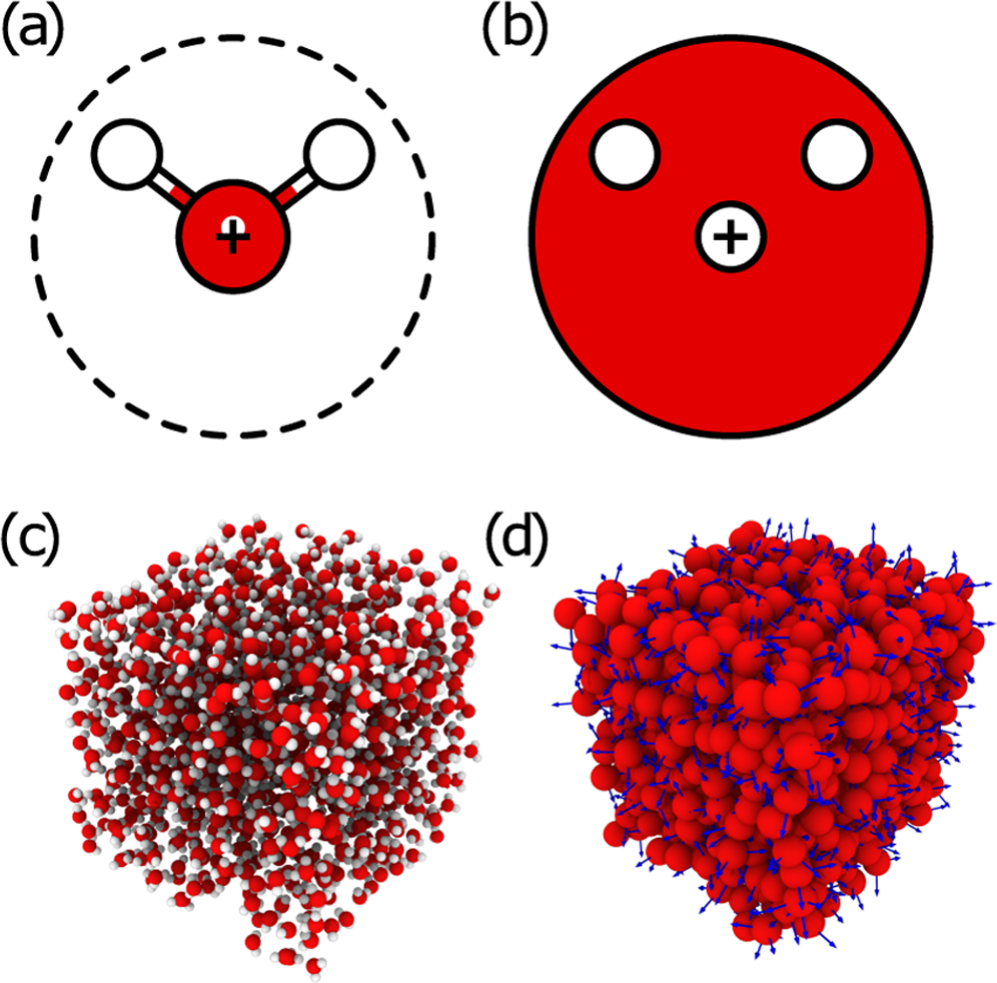
Schematic view of the (a) AA and (b) CG water models. The dashed line in (a) represents the Lennard-Jones sphere centered at the oxygen atom, while the small white point is the center-of-mass of the molecule. The red circle in (b) represents a finite-size sphere centered at the cross, while the white dots represent the virtual charges. Pictorial view of an (c) AA and the equivalent (d) CG sample of bulk water. The arrows in (d) show the orientation of the three axes of inertia per each finite-size sphere representing a single water molecule.

The MOLC force field has been described in detail elsewhere.^16^ In the MOLC model, the electrostatic interactions are accounted for *via* virtual sites acting as point charges (see Figure 1a,b). The sites are placed within a skin distance from the external surface of a parent CG particle, i.e., an ellipsoid. For the specific case of water, we use spheres, which are a special class of ellipsoids. The short- and long-range interactions are evaluated with a custom algorithm based on the Coulomb pairwise summation in real space and a particle–particle particle-mesh (PPPM) Ewalds solver in the reciprocal space.^23^ Typically, a reduced set of charges is used to replace the complete set of all-atom charges. However, here, we use the three charges of the original water model without any further modification. The novelty introduced in the MOLC model is that the point charges are defined in the ellipsoid framework to which they are related. In other words, each ellipsoid is decorated with a set of charges whose position is defined with respect to the three principal axes of the ellipsoid. As the virtual sites are defined anywhere inside the ellipsoid, we refer to them as “off-center” charges. At each timestep, the Cartesian coordinates of each virtual charge are reconstructed by combining the position and orientation of the parent ellipsoidal particle. In this way, there is no need to keep track of the position of the virtual charges nor to integrate it explicitly.

**Figure 2:**
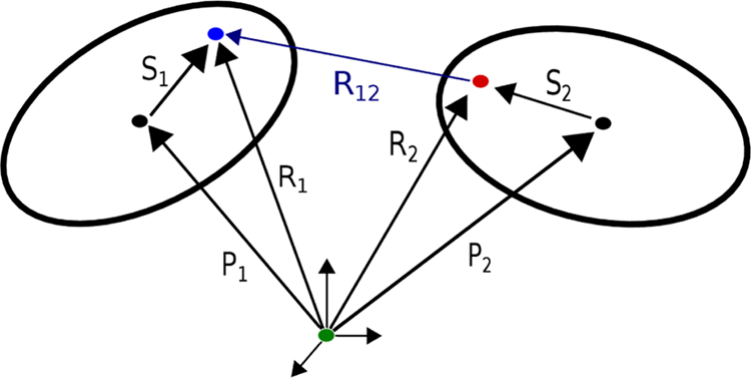
Diagram showing two off-center charges, in blue and red, defined for two ellipsoids.

Electrostatic interactions in the MOLC model are computed by rewriting the usual Coulombic expression from the charge frame of reference to the ellipsoid frame of reference. The direct Coulombic potential in real space is given by
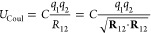
1where *C* is an energy-conversion constant, *q*_1_ and *q*_2_ are the virtual charges, and the norm  is the scalar distance. As shown in Figure 2, the vector distance between the virtual charges is expressed as
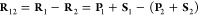
2where **R**_*i*_ = **P***_i_* + **S**_*i*_ (*i* = 1, 2) is the position of the virtual charge, **P***_i_* is the center of the ellipsoid, and **S**_*i*_ is the relative position of the virtual charges in the ellipsoid system of reference. In this notation, **R**_*i*_ is obtained by rotating the charge position from the ellipsoid’s frame to the frame of reference using the quaternion of the parent ellipsoid followed by a translation. The force that the virtual charge 1 exerts on its parent ellipsoid bead is computed from the gradient of the potential with respect to **P**_1_ as
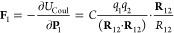
3while the force on the virtual charge 2 is simply defined as **F**_2_ = −**F**_1_. Finally, the torque is defined as
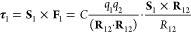
4while, in this case, the torque on the second particle can be written as
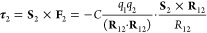
5All of the details of the derivation of forces and torques are reported in Supporting Information Note 1.

The AA water models studied are based on a three-site or four-site rigid molecule with a point charge on each site (in the case of Tip4P, the oxygen charge is displaced by 0.1546 Å), plus a single Lennard-Jones potential on the oxygen atom. The molecules are kept rigid with the SHAKE^24^ or RATTLE^25^ algorithms. In the MOLC force field, three atoms are replaced with a single sphere, whose mass is that of the water molecule and whose radius is the Lennard-Jones σ parameter. The virtual site representing the oxygen charge is placed at the center of the sphere, while the virtual sites representing the hydrogen atoms are placed on the plane *z* = 0 in the molecular frame of reference. Despite using one particle per molecule, the MOLC model of water includes all of the terms of the corresponding AA model, described with a different mathematical formalism. Consequently, an arbitrary AA configuration mapped to its CG counterpart will have the same intermolecular energy in both representations. A slight difference is expected for torques: in the AA model, each atom will generate a contribution acting on the center-of-mass of the rigid molecule; in the CG model, only the virtual particles carrying the charge of hydrogen atoms will produce a torque acting on the center of the CG sphere. However, this difference is limited as the center-of-mass of the water molecule lies some 0.07 Å from the position of oxygen, as shown in Figure 1a. The CG trajectories can be easily reverse-mapped to the AA representation using the position and quaternion of each ellipsoid, fully exploiting the extra rotational degrees of freedom associated with finite-size particles. The reverse-mapped trajectories were computed with the open-source Backmap code.^26^ For the special cases of the rigid water models (e.g., MD simulations integrated with the SHAKE algorithm^24^), the AA trajectories can be directly compared to the reverse-mapped trajectories using structural observables such as radial distribution functions (RDFs). All of the details related to the generation and equilibration of the simulated systems as well as the protocols adopted for the evaluation of the physical observables are reported in Supporting Information Note 2. Representative samples of the LAMMPS codes employed for the simulations are available in the Zenodo archive at https://doi.org/10.5281/zenodo.5552351.

## Results and Discussion

We test the validity of the MOLC representation for three widely used all-atom water models, namely, SPC-E,^27^ Tip3P-Ew,^28^ and Tip4P-05,^29^ by comparing the computed self-diffusion coefficient, dynamic viscosity, surface free energy, and enthalpy of vaporization^30−37^ between the AA and corresponding CG models at *T* = 298 K and *p* = 1 atm (see Supporting Information Note 2 for methodological details). For each AA and CG model, samples made of 500, 1000, and 5000 water molecules were studied to evaluate possible size dependence on the calculated physical observables.^38^ For the sake of simplicity, from this point on, we will refer to the Tip3P-Ew and Tip4P-05 models as Tip3P and Tip4P, respectively. A side-by-side comparison of AA and CG samples is shown in Figure 1c,d.

**Figure 3:**
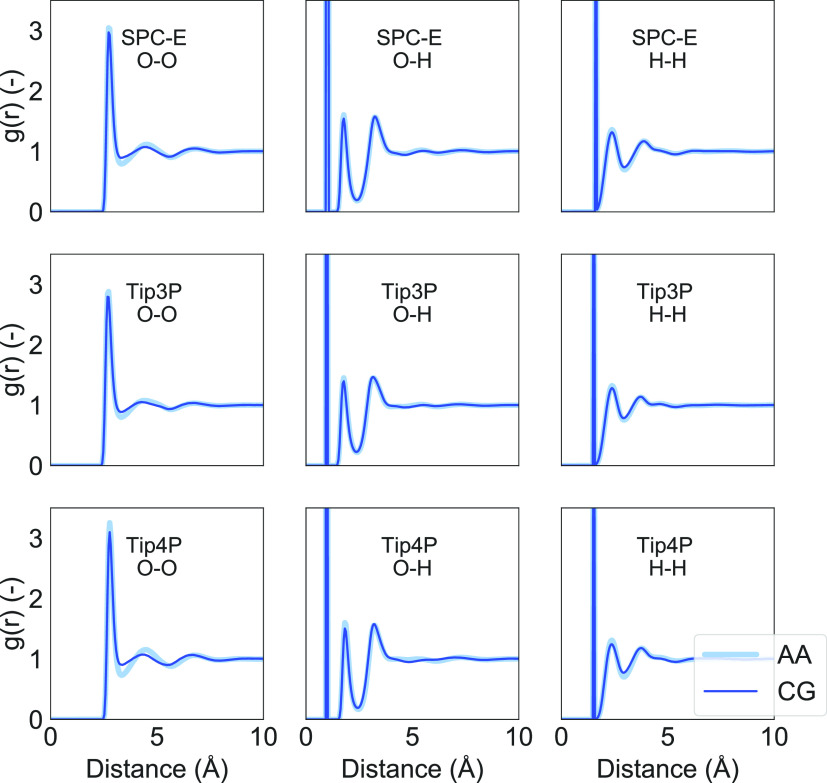
Radial distribution functions *g*(*r*) computed from all-atom (AA) and reverse-mapped coarse-grained (CG) samples for different water models and atoms.

The computed density of SPC-E and Tip4P CG models is 0.993 ± 0.004 g/cm^3^, while that of the Tip3P CG model is 0.995 ± 0.004 g/cm^3^, in excellent agreement with the corresponding AA values (0.994 ± 0.003 g/cm^3^ for SPC-E and Tip4P and 0.992 ± 0.004 g/cm^3^ for Tip3P). Furthermore, the 1-to-1 correspondence between the CG and AA representations allows us to reintroduce the atomic detail into the CG trajectories without loss of structural information (and vice-versa): the resulting reverse-mapped trajectory was then used to compute the oxygen–oxygen (O–O), oxygen–hydrogen (O–H), and hydrogen–hydrogen (H–H) radial distribution functions (RDFs) for the CG samples. The overlap between the RDFs of the AA and CG models in Figure 3 further corroborates the structural equivalence of the two models. The position and intensity of RDF peaks from reverse-mapped CG trajectories closely match those from AA simulations. The first and second peaks in the O–O distance are consistently broader in the CG samples, resulting also in a smaller depth of the first well, which is more pronounced for the TIP4P CG model. We attribute this difference to the shifted reference of the axis of inertia, which is centered in the center-of-mass in the AA model (Figure 1a) while in the center of the bead in the CG one (Figure 1b). This leads to slightly different dynamics between the AA and CG models. Such a difference is magnified in the TIP4P model since it includes an additional interaction center producing a torque on each molecule.

**Figure 4:**
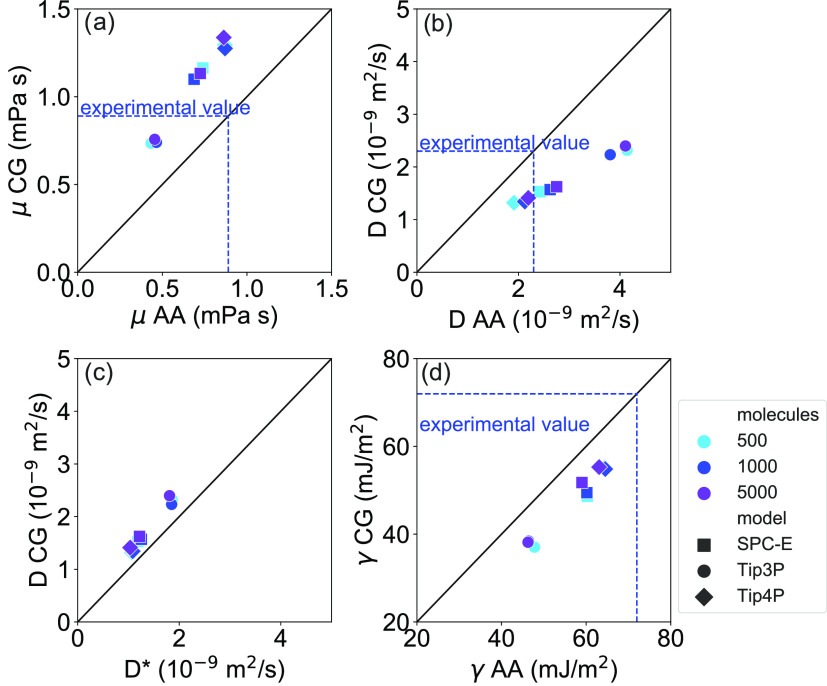
(a) Dynamic viscosity, (b) self-diffusion coefficient, and (d) surface free energy computed with an integration timestep of 1 fs for different CG and AA water models and system sizes. Error bars are reported in Supporting Information Table S1 for clarity. (c) Comparison between the self-diffusion coefficient calculated from the Stokes–Einstein relation () and from the mean square displacement (*D*) for the CG models. Water models are represented by different symbols, while system sizes are represented by colors. Experimental values refer to bulk water properties at *T* = 298 K and *p* = 1 atm.

The self-diffusion coefficient (*D*), dynamic viscosity (μ), and surface free energy (γ) of both the AA and CG water models evaluated with a timestep of 1 fs are summarized in Supporting Information Table S1. The results of AA simulations are in good agreement with reference modeling and experimental values found in the literature,^10,30,31,34,36^ without showing a system size dependence within the computed error bars (see Supporting Information Note 3 for details). Nevertheless, we observe a progressive reduction of the error bar of *D* with larger samples because of the improved statistics. The self-diffusion coefficient and dynamic viscosity derived from CG simulations are also plotted against the AA reference, as shown in Figure 4a,b. Results show that the self-diffusion coefficient computed from CG simulations is slightly lower than that from AA ones. Consistently, μ of CG samples is higher than that of AA ones. This evidence agrees with the Stokes–Einstein relation^31^ (, where *k*_B_ is the Boltzmann constant and *r* is the radius of water molecules, strictly valid for spherical particles), thus proving the self-consistency of the proposed model (see Figure 4c). The transient values of μ and *D* are reported in Supporting Information Figures S2 and S3, highlighting the substantial convergence of simulations. Furthermore, all obtained results remain in good agreement with the experimental values.^30,39^ Finally, in Figure 4d, we compare γ computed with the AA and CG models. The results show an average reduction with respect to the AA reference. However, in the specific case of SPC-E and Tip4P CG models, the calculated γ still represents a good approximation of the experimental value (72.0 mJ/m^2^),^40^ making the CG water models perfectly suitable for describing multiphase systems and interfacial phenomena. The observed differences between the AA and CG results can be explained by the fact that the moment of inertia of the CG model, based on a finite-size sphere, is about 10 times larger than that of the AA model.

**Figure 5:**
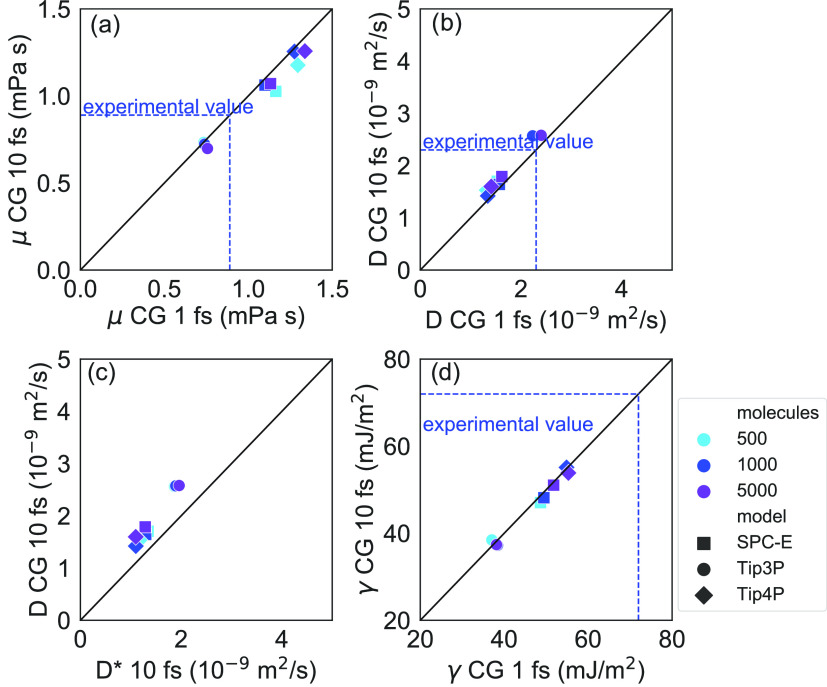
(a) Dynamic viscosity, (b) self-diffusion coefficient, and (d) surface free energy computed with integration timesteps of 1 fs and 10 fs for different CG models and system sizes. Error bars are reported in Supporting Information Table S2 for clarity. (c) Comparison between the self-diffusion coefficient calculated from the Stokes–Einstein relation () and from the mean square displacement (*D*) for the CG models with a 10 fs timestep. Water models are represented by different symbols, while system sizes are represented by colors. Experimental values refer to bulk water properties at *T* = 298 K and *p* = 1 atm.

The self-diffusion coefficient, dynamic viscosity, and surface free energy computed from the CG-MD simulations integrated with a longer timestep of 10 fs are summarized in Figure 5 and fully reported in Supporting Information Table S2. Also, in this case, the evaluated physical observables are not sensibly dependent on the system size. The viscosity and self-diffusion coefficients obtained with a longer timestep (Figure 5a,b) are consistent with those obtained with a timestep equal to 1 fs. The self-diffusion coefficients D* computed *via* the Stokes–Einstein relation from the μ values are also consistent with those obtained with the mean square displacement (Figure 5c). No significant difference is observed for the computed value of the surface free energy (Figure 5d). These results show that no substantial degradation of the computed properties of CG models is observed using a longer timestep of 10 fs. Furthermore, for the specific case of SPC-E and Tip4P CG models, we recall the good accuracy with respect to the experimental values.

The computed enthalpy of vaporization for samples of 5000 molecules is reported in Supporting Information Table S3, showing a substantial agreement between the AA and CG models. A comprehensive assessment of other thermodynamic properties, such as the low-temperature density anomaly or the melting temperature,^19,41,42^ will be the subject of future investigations.

**Figure 6:**
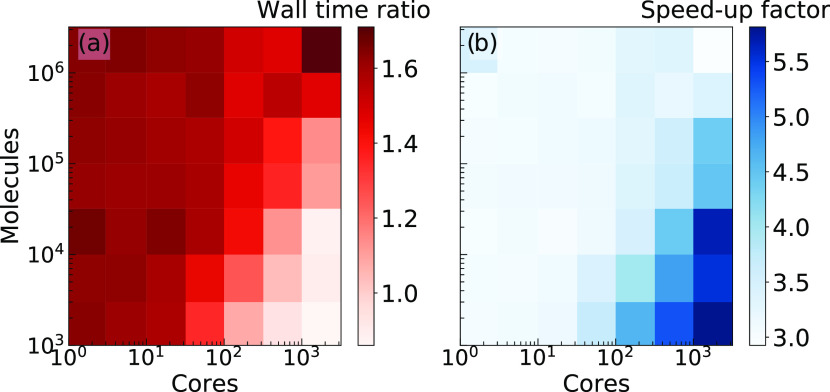
(a) Wall time ratio between the MOLC CG model and the corresponding AA model considering different boxes of Tip3P water molecules and a variable amount of computational cores (2 × 24 cores/node Intel Xeon 8160 at 2.10 GHz), but a fixed timestep of 1 fs for both models. (b) Speed-up factor of the CG model with respect to AA one, when the former is simulated with a timestep of 10 fs and the latter of 1 fs.

Considering the Tip3P water as a representative case study for assessing the computational performance of the MOLC model, Figure 6a reports the wall time ratio (wall time ratio = CG elapsed computational time/AA elapsed computational time) between CG and AA simulations with the same trajectory length. At a given timestep (1 fs), the computational cost of the CG models is approximately the same as the AA ones, the wall time ratio between CG and AA being in the range of 0.8–1.8. Indeed, despite reducing the number of particles from 3 to 1, the electrostatic interactions are identical in the AA and CG force fields and so is the computational cost. On the other hand, the possibility of using a timestep of 10 fs leads to a speed-up factor (speed-up factor = CG performance/AA performance, the performance being evaluated as nanoseconds of trajectory computed per day) of up to 6× (see Figure 6b).

The use of a longer timestep has the obvious advantage of significantly speeding up the mesoscopic model with respect to the original atomistic one. As a matter of fact, the timestep of AA water simulations typically does not exceed 2 fs, even for accelerated MD simulations of biomolecules where the scale of microseconds is routinely achieved.^43,44^ Here, we investigate the longest timestep still able to guarantee stable CG simulations and realistic water properties. For this, we compute the viscosity of the Tip3P water model using timesteps of 1, 10, 15, 20, 40, and 80 fs. As the timestep is increased, numerical instabilities arise from the accumulation of integration errors. To mitigate the inaccuracies in sampling the higher frequency motions, the mass of CG water molecules is progressively increased as to have a larger moment of inertia and slower dynamics (see Supporting Information Figure S4), an expedient already adopted in other works.^45^ The higher mass of CG water molecules leads to simulation stability of up to 80 fs timestep, but results show a steady increase of dynamic viscosity with mass (see Supporting Information Figure S5) in agreement with literature values.^46^ The scaled density and RDFs, instead, are in excellent agreement with the reference values even with the largest timestep tested.

A core feature of the MOLC model is to represent the intermolecular interactions via a Gay–Berne potential and point charges, which can be seamlessly mixed with standard AA force fields based on Lennard-Jones potentials. Using this framework, water models based on four or more point charges can be readily transformed into a CG model without any reparameterization, and AA force fields can be mixed with CG models in the same simulation. Another direction for the fine-tuning of CG water models involves replacing the finite-size isotropic spheres with finite-size anisotropic ellipsoids to reproduce the anisotropic inertia tensor of the AA water model. Ultra-coarse-grained models,^47−49^ i.e., where a single particle represents a cluster of water molecules, are also natively supported by the MOLC model and easily implementable by placing many electrostatic sites inside a large spheroidal particle, whose position and strength should be optimized to simultaneously reproduce the packing of pure water and its enthalpy of vaporization. The implementation of new water models is beyond the scope of this article but it is mentioned to encourage the translation of existing force fields in the MOLC representation. Similarly, from an application point of view, further studies will be necessary to validate the proposed model in multiphase systems (e.g., for the description of wettability or adsorption phenomena),^50−52^ nanoconfined geometries,^53,54^ or heat-transfer processes.^55,56^

## Conclusions

In this work, we have proposed, tested, and validated a new coarse description of classic water models based on the MOLC force field. We chose three popular all-atom models (SPC-E, Tip3P-Ew, and Tip4P-05) and found that their corresponding coarse-grained representations show accurate structural and dynamical properties: density, radial distribution functions, self-diffusion coefficient, dynamic viscosity, surface free energy, and enthalpy of vaporization. We observed a reduction of the CG self-diffusion coefficient, which is matched by an increase in the dynamic viscosity, consistent with the Stokes–Einstein relation. The computed surface free energy is approximately 14% smaller for all of the CG models. However, the computed surface free energy is still reasonably close to the experimental value, confirming that the CG models of water are good enough for describing interface problems such as material wettability or adsorption phenomena. A speed-up factor between 3 and 6 times is obtained with respect to the AA model, entirely being due to the increase in the integration timestep unlocked by coarse graining.

## Supporting Information Available

Derivation of forces and torques in the Molc model (Note 1), methodological detail (Note 2), detail for the evaluation of error bars (Note 3), Tables S1–S3, and Figures S1–S5 (PDF)

## References

[ref1] Kmiecik S.; Gront D.; Kolinski M.; Wieteska L.; Dawid A. E.; Kolinski A. Coarse-Grained Protein Models and Their Applications. Chem. Rev. 2016, 116, 7898–7936. 10.1021/acs.chemrev.6b00163. 27333362

[ref2] Ingólfsson H. I.; Lopez C. A.; Uusitalo J. J.; de Jong D. H.; Gopal S. M.; Periole X.; Marrink S. J. The power of coarse graining in biomolecular simulations. Wiley Interdiscip. Rev.: Comput. Mol. Sci. 2014, 4, 225–248. 10.1002/wcms.1169. 25309628 PMC4171755

[ref3] Brini E.; Algaer E. A.; Ganguly P.; Li C.; Rodríguez-Ropero F.; van der Vegt N. F. A. Systematic coarse-graining methods for soft matter simulations a review. Soft Matter 2013, 9, 2108–2119. 10.1039/C2SM27201F.

[ref4] Joshi S. Y.; Deshmukh S. A. A review of advancements in coarse-grained molecular dynamics simulations. Mol. Simul. 2020, 47, 786–803. 10.1080/08927022.2020.1828583.

[ref5] Srivastava R.; Fasano M.; Nejad S. M.; Thielemann H. C.; Chiavazzo E.; Asinari P. Carbon-Based Smart Materials; Charitidis C. A.; Koumoulos E. P.; Dragatogiannis D. A., Eds.; De Gruyter: Berlin/Boston, 2020; Chapter 3, pp 33–80, https://doi.org/10.1515/9783110479133-003.

[ref6] Yoshizawa T.; et al. Microsecond-timescale MD simulation of EGFR minor mutation predicts the structural flexibility of EGFR kinase core that reflects EGFR inhibitor sensitivity. npj Precis. Oncol. 2021, 5, 32 10.1038/s41698-021-00170-7. 33863983 PMC8052404

[ref7] Quinn T. R.; Steussy C. N.; Haines B. E.; Lei J.; Wang W.; Sheong F. K.; Stauffacher C. V.; Huang X.; Norrby P.-O.; Helquist P.; Wiest O. Microsecond timescale MD simulations at the transition state of PmHMGR predict remote allosteric residues. Chem. Sci. 2021, 12, 6413–6418. 10.1039/D1SC00102G. 34084441 PMC8115266

[ref8] Marrink S. J.; Risselada H. J.; Yefimov S.; Tieleman D. P.; de Vries A. H. The MARTINI Force Field: Coarse Grained Model for Biomolecular Simulations. J. Phys. Chem. B 2007, 111, 7812–7824. 10.1021/jp071097f. 17569554

[ref9] Yesylevskyy S. O.; SchÃfer L. V.; Sengupta D.; Marrink S. J. Polarizable Water Model for the Coarse-Grained MARTINI Force Field. PLoS Comput. Biol. 2010, 6, e1000810 10.1371/journal.pcbi.1000810. 20548957 PMC2883601

[ref10] Orsi M. Comparative assessment of the ELBA coarse-grained model for water. Mol. Phys. 2014, 112, 1566–1576. 10.1080/00268976.2013.844373.

[ref11] Riniker S.; van Gunsteren W. F. A simple, efficient polarizable coarse-grained water model for molecular dynamics simulations. J. Chem. Phys. 2011, 134, 084110 10.1063/1.3553378. 21361530

[ref12] Wu Z.; Cui Q.; Yethiraj A. A New Coarse-Grained Model for Water: The Importance of Electrostatic Interactions. J. Phys. Chem. B 2010, 114, 10524–10529. 10.1021/jp1019763. 20701383

[ref13] Cisneros G. A.; Wikfeldt K. T.; Ojamae L.; Lu J.; Xu Y.; Torabifard H.; Bartok A. P.; Csanyi G.; Molinero V.; Paesani F. Modeling Molecular Interactions in Water: From Pairwise to Many-Body Potential Energy Functions. Chem. Rev. 2016, 116, 7501–7528. 10.1021/acs.chemrev.5b00644. 27186804 PMC5450669

[ref14] Nguyen T. D.; Plimpton S. J. Aspherical particle models for molecular dynamics simulation. Comput. Phys. Commun. 2019, 243, 12–24. 10.1016/j.cpc.2019.05.010.

[ref15] Brown W. M.; Petersen M. K.; Plimpton S. J.; Grest G. S. Liquid crystal nanodroplets in solution. J. Chem. Phys. 2009, 130, 044901 10.1063/1.3058435. 19191407

[ref16] Ricci M.; Roscioni O. M.; Querciagrossa L.; Zannoni C. MOLC. A reversible coarse grained approach using anisotropic beads for the modelling of organic functional materials. Phys. Chem. Chem. Phys. 2019, 21, 26195–26211. 10.1039/C9CP04120F. 31755499

[ref17] Plimpton S. Fast Parallel Algorithms for Short-Range Molecular Dynamics. J. Comput. Phys. 1995, 117, 1–19. 10.1006/jcph.1995.1039.

[ref18] LAMMPS Molecular Dynamics Simulator, 2021. https://www.lammps.org.

[ref19] Lu J.; Qiu Y.; Baron R.; Molinero V. Coarse-Graining of TIP4P/2005, TIP4P-Ew, SPC/E, and TIP3P to Monatomic Anisotropic Water Models Using Relative Entropy Minimization. J. Chem. Theory Comput. 2014, 10, 4104–4120. 10.1021/ct500487h. 26588552

[ref20] Jin J.; Han Y.; Pak A. J.; Voth G. A. A new one-site coarse-grained model for water: Bottom-up many-body projected water (BUMPer). I. General theory and model. J. Chem. Phys. 2021, 154, 044104 10.1063/5.0026651. 33514116 PMC7826168

[ref21] Pluhackova K.; Backmann R. A. Biomembranes in atomistic and coarse-grained simulations. J. Phys.: Condens. Matter 2015, 27, 323103 10.1088/0953-8984/27/32/323103. 26194872

[ref22] Richardson L. F. IX; Glazebrook R. T. The approximate arithmetical solution by finite differences of physical problems involving differential equations, with an application to the stresses in a masonry dam. Philos. Trans. R. Soc., A 1911, 210, 307–357. 10.1098/rsta.1911.0009.

[ref23] Hockney R. W.; Eastwood J. W. Computer Simulation Using Particles; Adam Hilger: New York, 1989.

[ref24] Ryckaert J.-P.; Ciccotti G.; Berendsen H. J. Numerical integration of the cartesian equations of motion of a system with constraints: molecular dynamics of n-alkanes. J. Comput. Phys. 1977, 23, 327–341. 10.1016/0021-9991(77)90098-5.

[ref25] Andersen H. C. Rattle: A velocity version of the shake algorithm for molecular dynamics calculations. J. Comput. Phys. 1983, 52, 24–34. 10.1016/0021-9991(83)90014-1.

[ref26] Backmap: An Open-Source Program to Reverse Map Coarse-Grained Trajectories to Atomic Coordinates, 2021. https://github.com/matteoeghirotta/backmap_legacy.

[ref27] Berendsen H. J. C.; Grigera J. R.; Straatsma T. P. The missing term in effective pair potentials. J. Phys. Chem. A. 1987, 91, 6269–6271. 10.1021/j100308a038.

[ref28] Price D. J.; Brooks C. L. 3rd A modified TIP3P water potential for simulation with Ewald summation. J. Chem. Phys. 2004, 121, 10096–10103. 10.1063/1.1808117. 15549884

[ref29] Abascal J. L. F.; Vega C. A general purpose model for the condensed phases of water: TIP4P/2005. J. Chem. Phys. 2005, 123, 234505 10.1063/1.2121687. 16392929

[ref30] González M. A.; Abascal J. L. F. The shear viscosity of rigid water models. J. Chem. Phys. 2010, 132, 096101 10.1063/1.3330544. 20210414

[ref31] Guevara-Carrion G.; Vrabec J.; Hasse H. Prediction of self-diffusion coefficient and shear viscosity of water and its binary mixtures with methanol and ethanol by molecular simulation. J. Chem. Phys. 2011, 134, 074508 10.1063/1.3515262. 21341860

[ref32] Zhang Y.; Otani A.; Maginn E. J. Reliable Viscosity Calculation from Equilibrium Molecular Dynamics Simulations: A Time Decomposition Method. J. Chem. Theory Comput. 2015, 11, 3537–3546. 10.1021/acs.jctc.5b00351. 26574439

[ref33] Guo G.-J.; Zhang Y.-G. Equilibrium molecular dynamics calculation of the bulk viscosity of liquid water. Mol. Phys. 2001, 99, 283–289. 10.1080/00268970010011762.

[ref34] Tsimpanogiannis I. N.; Moultos O. A.; Franco L. F. M.; Spera M. B. D. M.; Erdős M.; Economou I. G. Self-diffusion coefficient of bulk and confined water: a critical review of classical molecular simulation studies. Mol. Simul. 2019, 45, 425–453. 10.1080/08927022.2018.1511903.

[ref35] Vega C.; Abascal J. L. F.; Conde M. M.; Aragones J. L. What ice can teach us about water interactions: a critical comparison of the performance of different water models. Faraday Discuss. 2009, 141, 251–276. 10.1039/B805531A. 19227361

[ref36] Vega C.; de Miguel E. Surface tension of the most popular models of water by using the test-area simulation method. J. Chem. Phys. 2007, 126, 154707 10.1063/1.2715577. 17461659

[ref37] Wang J.; Hou T. Application of Molecular Dynamics Simulations in Molecular Property Prediction. 1. Density and Heat of Vaporization. J. Chem. Theory Comput. 2011, 7, 2151–2165. 10.1021/ct200142z. 21857814 PMC3156483

[ref38] Yeh I.-C.; Hummer G. System-Size Dependence of Diffusion Coefficients and Viscosities from Molecular Dynamics Simulations with Periodic Boundary Conditions. J. Phys. Chem. B 2004, 108, 15873–15879. 10.1021/jp0477147.

[ref39] Holz M.; Heil S. R.; Sacco A. Temperature-dependent self-diffusion coefficients of water and six selected molecular liquids for calibration in accurate 1H NMR PFG measurements. Phys. Chem. Chem. Phys. 2000, 2, 4740–4742. 10.1039/b005319h.

[ref40] Vargaftik N. B.; Volkov B. N.; Voljak L. D. International Tables of the Surface Tension of Water. J. Phys. Chem. Ref. Data 1983, 12, 817–820. 10.1063/1.555688.

[ref41] Lu J.; Chakravarty C.; Molinero V. Relationship between the line of density anomaly and the lines of melting, crystallization, cavitation, and liquid spinodal in coarse-grained water models. J. Chem. Phys. 2016, 144, 234507 10.1063/1.4953854. 27334179

[ref42] Vega C.; Abascal J. L. F. Simulating water with rigid non-polarizable models: a general perspective. Phys. Chem. Chem. Phys. 2011, 13, 19663–19688. 10.1039/c1cp22168j. 21927736

[ref43] Pierce L. C.; Salomon-Ferrer R.; Augusto F. de Oliveira C.; McCammon J. A.; Walker R. C. Routine Access to Millisecond Time Scale Events with Accelerated Molecular Dynamics. J. Chem. Theory Comput. 2012, 8, 2997–3002. 10.1021/ct300284c. 22984356 PMC3438784

[ref44] Juárez-Jiménez J.; Gupta A. A.; Karunanithy G.; Mey A. S. J. S.; Georgiou C.; Ioannidis H.; de Simone A.; Barlow P. N.; Hulme A. N.; Walkinshaw M. D.; Baldwin A. J.; Michel J. Dynamic design: manipulation of millisecond timescale motions on the energy landscape of cyclophilin A. Chem. Sci. 2020, 11, 2670–2680. 10.1039/C9SC04696H. 34084326 PMC8157532

[ref45] Orsi M.; Essex J. W. The ELBA Force Field for Coarse-Grain Modeling of Lipid Membranes. PLoS One 2011, 6, e28637 10.1371/journal.pone.0028637. 22194874 PMC3241685

[ref46] Chamorro V. C.; Tempra C.; Jungwirth P. Heavy Water Models for Classical Molecular Dynamics: Effective Inclusion of Nuclear Quantum Effects. J. Phys. Chem. B 2021, 125, 4514–4519. 10.1021/acs.jpcb.1c02235. 33904303

[ref47] He X.; Shinoda W.; DeVane R.; Klein M. L. Exploring the utility of coarse-grained water models for computational studies of interfacial systems. Mol. Phys. 2010, 108, 2007–2020. 10.1080/00268976.2010.503197.

[ref48] Li M.; Lu W.; Zhang J. Z. A three-point coarse-grained model of five-water cluster with permanent dipoles and quadrupoles. Phys. Chem. Chem. Phys. 2020, 22, 26289–26298. 10.1039/D0CP04782A. 33174895

[ref49] Li M.; Lu W.; Zhang J. Z. Ultra-coarse-graining modeling of liquid water. J. Chem. Phys. 2021, 154, 224506 10.1063/5.0055453. 34241208

[ref50] Goossens S.; Seveno D.; Rioboo R.; Vaillant A.; Conti J.; De Coninck J. Can We Predict the Spreading of a Two-Liquid System from the Spreading of the Corresponding Liquid-Air Systems?. Langmuir 2011, 27, 9866–9872. 10.1021/la200439e. 21682265

[ref51] Fasano M.; Falciani G.; Brancato V.; Palomba V.; Asinari P.; Chiavazzo E.; Frazzica A. Atomistic modelling of water transport and adsorption mechanisms in silicoaluminophosphate for thermal energy storage. Appl. Therm. Eng. 2019, 160, 114075 10.1016/j.applthermaleng.2019.114075.

[ref52] Fasano M.; Bevilacqua A.; Chiavazzo E.; Humplik T.; Asinari P. Mechanistic correlation between water infiltration and framework hydrophilicity in MFI zeolites. Sci. Rep. 2019, 9, 18429 10.1038/s41598-019-54751-5. 31804543 PMC6895097

[ref53] Chiavazzo E.; Fasano M.; Asinari P.; Decuzzi P. Scaling behaviour for the water transport in nanoconfined geometries. Nat. Commun. 2014, 5, 3565 10.1038/ncomms4565. PMC3988813 24699509

[ref54] Johansson P.; Hess B. Molecular origin of contact line friction in dynamic wetting. Phys. Rev. Fluids 2018, 3, 074201 10.1103/PhysRevFluids.3.074201.

[ref55] Tascini A. S.; Armstrong J.; Chiavazzo E.; Fasano M.; Asinari P.; Bresme F. Thermal transport across nanoparticle-fluid interfaces: the interplay of interfacial curvature and nanoparticle-fluid interactions. Phys. Chem. Chem. Phys. 2017, 19, 3244–3253. 10.1039/C6CP06403E. 28083587

[ref56] Yd S.; Maroo S. C. Surface-heating algorithm for water at nanoscale. J. Phys. Chem. Lett. 2015, 6, 3765–3769. 10.1021/acs.jpclett.5b01627. 26722754

