# Supplementary material for: Anisotropic Electrostatic Interactions in Coarse-Grained Water Models to Enhance the Accuracy and Speed-Up Factor of Mesoscopic Simulations
Source: J Phys Chem B. 2021 Oct 27;125(43):12020–7. doi: 10.1021/acs.jpcb.1c07642 (PMC8573754; doi:10.1021/acs.jpcb.1c07642)
Supplement: Supplementary file 1 — jp1c07642_si_001.pdf [file jp1c07642_si_001.pdf]

Anisotropic Electrostatic Interactions in Coarse-Grained Water Models to Enhance the Accuracy and Speed-up Factor of Mesoscopic Simulations

# – Supporting Information –

Francesco Maria Bellussi,† Otello Maria Roscioni,*,‡ Matteo Ricci,‡ and Matteo Fasano*,†

†*Department of Energy, Politecnico di Torino, Torino, Italy*

‡*MaterialX LTD, Bristol, UK*

E-mail: om.roscioni@materialx.co.uk; matteo.fasano@polito.it

In this document we provide supporting notes, tables and figures with additional details on model development and obtained results.

Supporting Note 1: Derivation of Forces and Torques in the Molc Model

As reported also in the main manuscript, the direct Coulomb potential in real space is given by:

$$U_{Coul} = C \cdot \frac{q_1\ q_2}{R_{12}} = C \cdot \frac{q_1\ q_2}{\sqrt{\mathbf{R}_{12} \cdot \mathbf{R}_{12}}}, \tag{S1}$$

where C is an energy–conversion constant, q_1 and q_2 the virtual charges and the norm $R_{12} = \sqrt{\mathbf{R}_{12} \cdot \mathbf{R}_{12}}$ is the scalar distance. The vector distance between the virtual charges is expressed as:

$$\mathbf{R}_{12} = \mathbf{R}_1 - \mathbf{R}_2 = \mathbf{P}_1 + \mathbf{S}_1 - (\mathbf{P}_2 + \mathbf{S}_2), \tag{S2}$$

where $\mathbf{R}_i = \mathbf{P}_i + \mathbf{S}_i$ ($i = 1,\ 2$) is the position of the virtual charge, $\mathbf{P}_i$ the centre of the ellipsoid, and $\mathbf{S}_i$ the relative position of the virtual charges in the ellipsoid system of reference. In this notation, $\mathbf{R}_i$ is obtained by rotating the charge position from the ellipsoid's frame to the frame of reference using the quaternion of the parent ellipsoid followed by a translation, as shown in Figure 1 in the manuscript. The dot product in Equation S1 can be expanded as follows:

$$\begin{aligned}\mathbf{R}_{12} \cdot \mathbf{R}_{12} = {} & \mathbf{P}_1 \cdot \mathbf{P}_1 + \mathbf{S}_1 \cdot \mathbf{S}_1 + \mathbf{P}_2 \cdot \mathbf{P}_2 + \mathbf{S}_2 \cdot \mathbf{S}_2 + 2\ \mathbf{P}_1 \cdot \mathbf{S}_1 + 2\ \mathbf{P}_2 \cdot \mathbf{S}_2 \\ & - 2\ \mathbf{P}_1 \cdot \mathbf{P}_2 - 2\ \mathbf{S}_1 \cdot \mathbf{S}_2 - 2\ \mathbf{P}_1 \cdot \mathbf{S}_2 - 2\ \mathbf{P}_2 \cdot \mathbf{S}_1.\end{aligned} \tag{S3}$$

The force that the virtual charge 1 exerts on its parent ellipsoid bead is computed from the gradient of the potential with respect to $\mathbf{P}_1$. The derivative $\partial(\mathbf{R}_{12} \cdot \mathbf{R}_{12})/\partial\mathbf{P}_1$ is calculated below:

$$\frac{\partial(\mathbf{R}_{12} \cdot \mathbf{R}_{12})}{\partial \mathbf{P}_1} = 2\ \mathbf{P}_1 + 0 + 0 + 0 + 2\ \mathbf{S}_1 + 0 - 2\ \mathbf{P}_2 - 0 - 2\ \mathbf{S}_2 - 0 = 2\ \mathbf{R}_{12}. \tag{S4}$$

Hence the force becomes:

$$
\begin{aligned}
\mathbf{F}_1 = -\frac{\partial U_{Coul}}{\partial \mathbf{P}_1} &= C \cdot \left[\frac{q_1\ q_2}{\sqrt{\mathbf{R}_{12} \cdot \mathbf{R}_{12}}} \right]'_{\mathbf{P}_1} \\
&= -C\ \frac{q_1\ q_2}{-2\ \sqrt{\mathbf{R}_{12} \cdot \mathbf{R}_{12}}\ (\mathbf{R}_{12} \cdot \mathbf{R}_{12})} [\mathbf{R}_{12} \cdot \mathbf{R}_{12}]'_{\mathbf{P}_1} \\
&= C\ \frac{q_1\ q_2}{2\ \sqrt{\mathbf{R}_{12} \cdot \mathbf{R}_{12}}\ (\mathbf{R}_{12} \cdot \mathbf{R}_{12})} \cdot 2\ \mathbf{R}_{12} \\
&= C\ \frac{q_1\ q_2}{(\mathbf{R}_{12} \cdot \mathbf{R}_{12})} \cdot \frac{\mathbf{R}_{12}}{\sqrt{\mathbf{R}_{12} \cdot \mathbf{R}_{12}}} \\
&= C\ \frac{q_1\ q_2}{(\mathbf{R}_{12} \cdot \mathbf{R}_{12})} \cdot \frac{\mathbf{R}_{12}}{R_{12}}
\end{aligned} \tag{S5}
$$

while the force on the virtual charge 2 is simply defined as $\mathbf{F}_2 = -\mathbf{F}_1$. The torque is defined as:

$$
\tau_1 = \mathbf{S}_1 \times \mathbf{F}_1 = C\ \frac{q_1\ q_2}{(\mathbf{R}_{12} \cdot \mathbf{R}_{12})} \cdot \frac{\mathbf{S}_1 \times \mathbf{R}_{12}}{R_{12}}. \tag{S6}
$$

In this case the torque on the second particle can be written as:

$$
\tau_2 = \mathbf{S}_2 \times \mathbf{F}_2 = -C\ \frac{q_1\ q_2}{(\mathbf{R}_{12} \cdot \mathbf{R}_{12})} \cdot \frac{\mathbf{S}_2 \times \mathbf{R}_{12}}{R_{12}}. \tag{S7}
$$

The calculation of electrostatic interactions *via* virtual charges has been implemented in a user-defined package for the program LAMMPS[1,2] and can be accessed via the keywords `pair_style coul/long/offcentre` and `kspace_style pppm/offcentre`. The code is freely available on GitHub.[3]

Supporting Note 2: Methodological detail

The initial configuration of each simulated system was generated using the program Moltemplate,[4] with 3D periodic boundary conditions (PBC) applied. A multistage compression was then performed at 298 K by shrinking the initial simulation box until the system reached a target density of 1 g/cm^3. At each step, the box volume was reduced by 14% every 50 ps. Finally, the system was left free to relax in the NPT ensemble at 1 atm and 298 K for 4 ns (Figure S1). The van der Waals and electrostatic interactions were modelled with

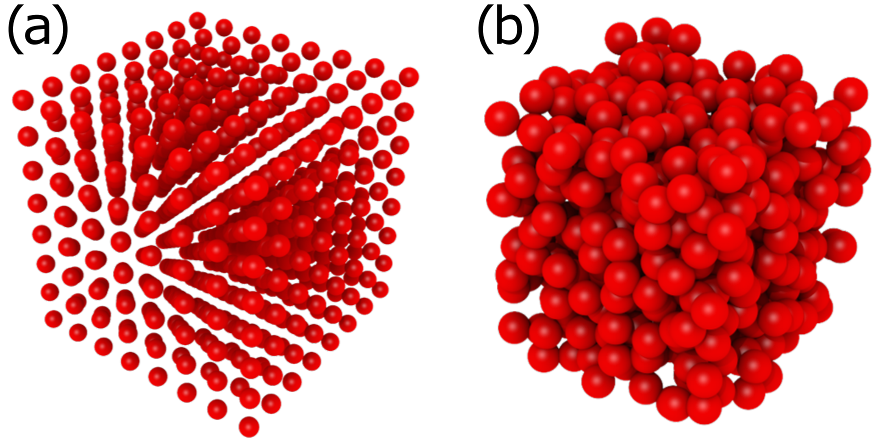

Figure S1: (a) Initial guess and (b) condensed state at 298 K and 1 atm for a sample of 1000 CG water molecules.

a cut-off radius of 12 Å and 10 Å, respectively. The long-range electrostatic interactions were calculated with the particle-particle particle-mesh (PPPM) method.[5] The all-atoms MD simulations were integrated using a timestep of 1 fs, while timesteps of 1 fs or 10 fs were used for the CG simulations.

The dynamic viscosity η was calculated from the auto-correlation function (ACF) of the off-diagonal components of the stress tensor, as expressed by the Green-Kubo relation:[6]

$$\eta = \frac{V}{k_B T} \int_0^{\infty} \langle P_t(0) \cdot P_t(t) \rangle dt, \tag{S8}$$

where k_B is the Boltzmann constant, V the volume, T the absolute system temperature

and P_t the transverse interface pressure, while the brackets indicate an ensemble average and $\cdot$ a dot product. The ACF is calculated every 2 fs with an integration timestep of 1 fs, and the upper limit of the integral was defined at 8 ps and 16 ps in the case of the AA and CG simulations, respectively. In the CG simulations with an integration timestep of 10 fs, the ACF is calculated every 20 fs, leaving the upper limit at 16 ps. To obtain reliable statistics, simulations were performed considering the average of the partial contributions in each orthogonal direction along 20 ns of trajectories after 20 ns of stabilization. The error bars were evaluated combining both the space and time standard deviations. The upper limit for the AA simulation was chosen to increase the accuracy of previous work available in the literature,[7] even if we did not observe significant differences after reaching the plateau (see Figure S2). On the other hand, a larger integral upper limit for CG simulations was chosen to avoid possible effects of the ACF tail, and to obtain a good confidence interval for the final value.

The self-diffusion coefficient D was calculated from the mean square displacement (MSD) of water particles as a function of the observation time using the classical Einstein relation:[8]

$$D = \frac{1}{2d} \lim_{t \to \infty} \frac{\langle [r(t) - r(0)]^2 \rangle}{t}, \tag{S9}$$

where d is the dimensionality of the system, t the observation time and $\langle [r(t) - r(0)]^2 \rangle$ the system averaged mean square displacement. The MSD was evaluated as the space average of each orthogonal direction and the time average of 5 equally spaced blocks, along a trajectory of 4 ns (see Figure S3). Also in this case, the error bars were evaluated combining both the space and time standard deviations.

The surface free energy γ was evaluated using the Kirkwood-Buff relation on a slab geometry. This relation is based on the normal and tangential pressure difference due to the formation of the surface:[9]

$$\gamma = \frac{L_z}{2} \left[\langle P_z \rangle - \frac{\langle P_x \rangle + \langle P_y \rangle}{2} \right], \tag{S10}$$

where P_z is the pressure in the direction orthogonal to the surface, P_x and P_y are the pressures in the transverse directions, and L_z is the length of the simulation box in the orthogonal direction to the surface. γ was evaluated along a trajectory of 5 ns, as the average of 5 equally spaced blocks, after 5 ns of stabilization. The slab geometry was generated using PBC along x and y, and non-PBC along z, applying the slab correction for the long-range interactions.[10] A reflective wall was applied to prevent atoms or particles to migrate beyond the z fixed boundary direction.

The enthalpy of vaporisation of pure water was evaluated for AA and CG samples of 5000 water molecules using the textbook definition:[11]

$$\Delta H_{\mathrm{vap}}(T) = E^{\mathrm{inter}}_{\mathrm{liquid}}(T) + RT \tag{S11}$$

where $E^{\mathrm{inter}}_{\mathrm{liquid}}(T)$ is the inter-molecular energy of liquid water at a given T.

The results of dynamic viscosity, self-diffusion coefficient, surface tension and enthalpy of vaporization are summarized in Tables S1, S2 and S3.

Supporting Note 3: Detail for the evaluation of error bars

The error bars of the dynamic viscosity and self-diffusion coefficient were calculated considering the averaged value in time and space. Firstly we evaluated the mean value with its standard deviation in time for each orthogonal direction. Then we evaluated the mean ($\overline{X}$) and standard deviation (σ) of the three orthogonal directions ($k = 3$) mean values as:

$$\overline{X} = \frac{\sum_{i=1}^{k} \overline{x_i}}{k} \tag{S12}$$

$$\sigma^2 = \frac{\sum_{i=1}^{k}[\sigma_i^2(n_i - 1)] + \sum_{i=1}^{k}[n_i(\overline{x_i} - \overline{X})^2]}{\sum_{i=1}^{k} n_i - 1}, \tag{S13}$$

where $\overline{x_i}$ is the time average in each orthogonal direction and $\overline{\sigma_i}$ its corresponding standard deviation, n_i is the total number of time blocks for each orthogonal direction.

Supporting Tables: Summarized results

Table S1: Self-diffusion coefficient (D, 10^{-9} m^2/s), dynamic viscosity (μ, mPa s) and surface free energy (γ, mJ/m^2) considering a timestep of 1 fs. Results from all-atoms (AA) and coarse-grained (CG) simulations are reported.

	Mol	D AA	D CG 1fs	μ AA	μ CG 1 fs	γ AA	γ CG 1fs
SPC-E	500	2.42±0.74	1.53±0.14	0.74±0.01	1.16±0.04	60.28±2.08	48.64±1.26
	1000	2.62±0.29	1.57±0.19	0.69±0.01	1.10±0.04	59.45±0.47	49.09±1.55
	5000	2.75±0.16	1.62±0.13	0.72±0.02	1.13±0.03	59.02±1.12	51.77±1.80
Tip3P	500	4.14±0.82	2.32±0.44	0.43±0.02	0.74±0.03	47.80±0.85	37.05±1.88
	1000	3.81±0.57	2.23±0.25	0.46±0.01	0.74±0.03	46.40±0.98	38.41±1.37
	5000	4.11±0.33	2.40±0.16	0.45±0.01	0.76±0.02	46.26±0.38	38.16±0.63
Tip4P	500	1.91±0.41	1.32±0.34	0.87±0.03	1.30±0.05	64.38±2.37	55.16±1.59
	1000	2.13±0.32	1.34±0.16	0.87±0.01	1.28±0.04	64.52±0.98	54.84±1.51
	5000	2.19±0.11	1.41±0.08	0.86±0.02	1.34±0.03	63.07±1.54	55.29±0.78

Table S2: Self-diffusion coefficient (D, 10^{-9} m^2/s), dynamic viscosity (μ, mPa s) and surface free energy (γ, mJ/m^2) considering a timestep of 10 fs. Results from coarse-grained (CG) simulations are reported.

	Mol	D CG 10fs	μ CG 10fs	γ CG 10fs
SPC-E	500	1.70±0.28	1.03±0.06	47.05±2.05
	1000	1.64±0.25	1.06±0.05	48.15±1.54
	5000	1.79±0.11	1.07±0.05	51.05±1.63
Tip3P	500	2.56±0.41	0.73±0.04	38.47±1.98
	1000	2.57±0.39	0.72±0.01	37.25±1.18
	5000	2.58±0.16	0.70±0.02	37.39±1.07
Tip4P	500	1.53±0.23	1.18±0.02	53.92±1.92
	1000	1.42±0.21	1.26±0.03	55.12±2.71
	5000	1.60±0.13	1.26±0.05	53.87±1.78

Table S3: Enthalpy of vaporization (ΔH_{vap}, kJ/mol). Results from all-atoms (AA) and coarse-grained (CG) simulations are reported. The reference experimental value is 44 kJ/mol.[12]

Model	ΔH_{vap} AA	ΔH_{vap} CG
SPC-E	49.1±0.09	49.4±0.09
Tip3P	44.1±0.09	44.5±0.09
Tip4P	50.1±0.10	50.4±0.10

Supporting Figures: Additional Results

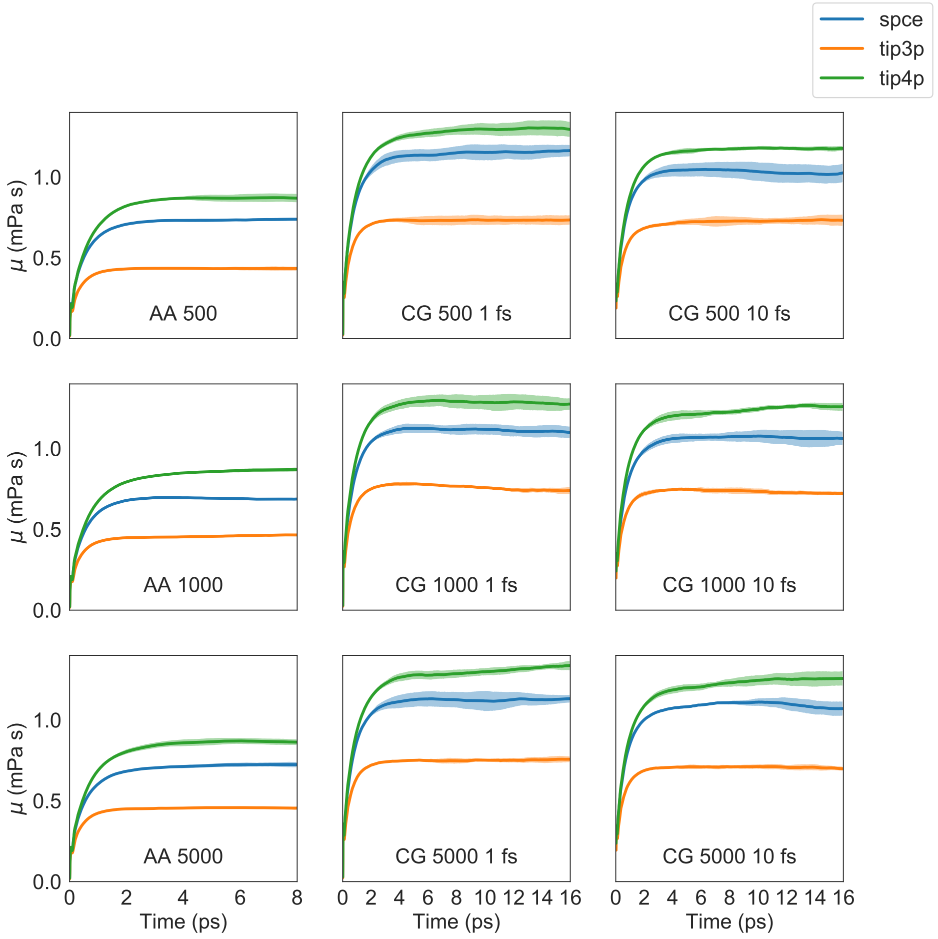

Figure S2: Integrated auto-correlation functions (ACF) for the evaluation of the dynamic viscosity of the system studied. The upper limit of the integral of the ACFs for the AA systems is fixed at 8 ps, while in the case of the CG systems is at 16 ps, to evaluate possible tail effects. The error bands are obtained considering the deviation with respect to the mean on three orthogonal dimensions and the mean value in time.

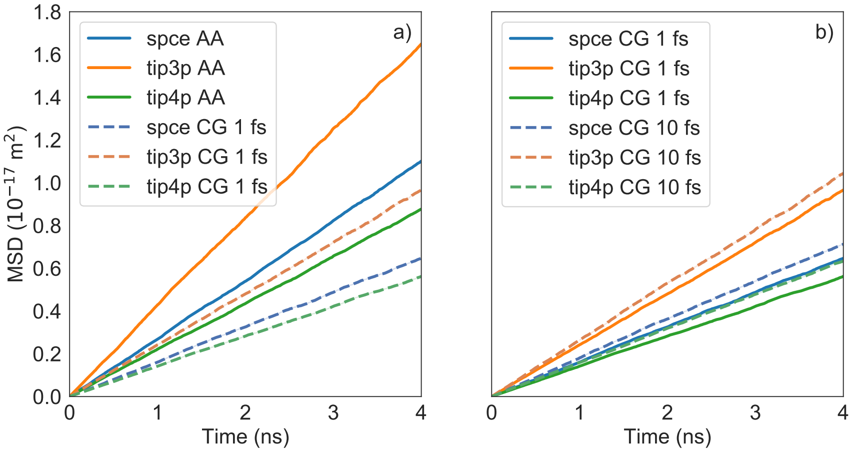

Figure S3: Mean square displacements of water molecules for the systems studied. (a) Comparison between AA and CG systems integrated with a time step of 1 fs. (b) Comparison between CG systems integrated with a timestep of 1 fs and 10 fs.

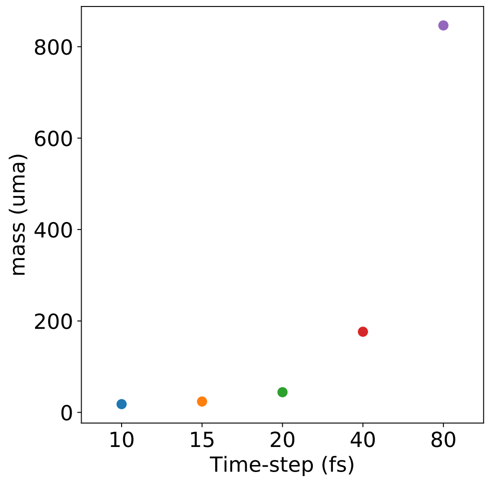

Figure S4: Variation of mass for the CG Tip3P water model to limit the numerical instabilities while increasing the integration timestep.

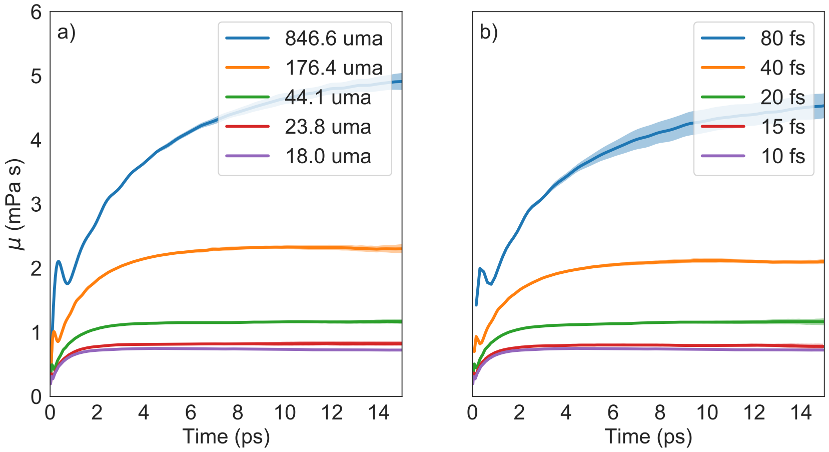

Figure S5: (a) Variation of dynamic viscosity for different water masses with an integration timestep of 10 fs and Tip3P CG model. (b) Variation of dynamic viscosity for different water masses and different timesteps, considering a Tip3P CG model. Comparing the two panels, we can observe that the dynamic viscosity depends mainly on the water mass and not on the integration timestep.

References

(1) Plimpton, S. Fast Parallel Algorithms for Short-Range Molecular Dynamics. *J. Comput. Phys.* **1995**, *117*, 1–19.

(2) LAMMPS Molecular Dynamics Simulator. https://www.lammps.org.

(3) USER-MOLC package is available on GitHub. https://github.com/matteoeghirotta/lammps-30Oct19.

(4) Jewett, A. I.; Stelter, D.; Lambert, J.; Saladi, S. M.; Roscioni, O. M.; Ricci, M.; Autin, L.; Maritan, M.; Bashusqeh, S. M.; Keyes, T.; Dame, R. T.; Shea, J.-E.; Jensen, G. J.; Goodsell, D. S. Moltemplate: A Tool for Coarse-Grained Modeling of Complex Biological Matter and Soft Condensed Matter Physics. *J. Mol. Biol.* **2021**, 166841.

(5) Hockney, R. W.; Eastwood, J. W. *Computer simulation using particles*; Adam Hilger: New York, 1989.

(6) Tuckerman, M. E. *Statistical mechanics theory and molecular simulation*; Oxford graduate texts; Oxford University Press: New York, 2010.

(7) González, M. A.; Abascal, J. L. F. The shear viscosity of rigid water models. *J. Chem. Phys.* **2010**, *132*, 096101.

(8) Allen, M.; Tildesley, D. *Computer Simulation of Liquids*; Oxford: Clarendon Pr, 1987.

(9) Kirkwood, J. G.; Buff, F. P. The Statistical Mechanical Theory of Surface Tension. *J. Chem. Phys.* **1949**, *17*, 338–343.

(10) Yeh, I.-C.; Berkowitz, M. L. Ewald summation for systems with slab geometry. *J. Chem. Phys.* **1999**, *111*, 3155–3162.

(11) Wang, J.; Hou, T. Application of Molecular Dynamics Simulations in Molecular Property Prediction. 1. Density and Heat of Vaporization. *J. Chem. Theory Comput.* **2011**, *7*, 2151–2165, PMID: 21857814.

(12) Vega, C.; Abascal, J. L. F. Simulating water with rigid non-polarizable models: a general perspective. *Phys. Chem. Chem. Phys.* **2011**, *13*, 19663–19688.
